# “Scientist as a Discoverer and Explorer of Unknown Lands”: Quo Vadis?

**DOI:** 10.1007/s12124-026-10021-4

**Published:** 2026-07-20

**Authors:** Joseph Glicksohn

**Affiliations:** 1 Department of Criminology, Bar-Ilan University, Ramat Gan, 52100 Israel; 2 The Leslie and Susan Gonda (Goldschmied) Multidisciplinary Brain Research Center, Bar-Ilan University, Ramat Gan, Israel

**Keywords:** Heinz Werner, Process, Contour formation, *Ganzfeld*, Hypothesis testing, Exploration

## Abstract

Heinz Werner contrasted the consensual preoccupation with hypothesis testing in experimental research and his own emphasis on the process of exploration. In this paper, the type of exploratory process that Werner is referring to is addressed, using his classic study on contour formation to exemplify this. In that study, Werner explored the phenomenon of contour formation, without testing any particular hypothesis. To further consider the distinction between the systematic exploration of a phenomenon, with a study designed to prove or disprove a specific hypothesis, I consider a study exemplifying the latter. I argue that Werner’s thinking has relevance for active researchers today and conclude that the present concern regarding the ‘replication crisis’ and the complementary advocacy of the pre-registration of studies is inherently detrimental to the process of exploration and discovery that Werner promoted for psychological research.

## Introduction: “Twenty-Five Years Ago”

While Heinz Werner (1890–1964) is indeed “a forgotten pioneer in developmental sciences (Selau & Fossa, 2026), for those who are familiar with his work and his legacy (for earlier reviews, see Franklin, 1990; Glick, 1992; Ostler, 2016), his thoughts about the conduct of research have an even greater urgency at the present, given our contemporary research climate promoting the pre-registration of studies, with a strict abidance to a pre-registered experimental protocol (e.g., Simmons et al., 2021–already cited 277 times, according to Google Scholar). Werner’s approach, as Franklin (1990, p. 185) writes, “was suspect” then (in the 30 s, 40 s, 50 s, and 60 s) “In an era when doing good science meant doing ‘hard’ science”, because “Both its theoretical presuppositions and methodological openness made it vulnerable to accusations of ‘softness.’” Werner advocated for the conduct of exploratory research, as opposed to one only concerned with hypothesis testing, and in this paper I will attempt to elaborate on what he meant by this, and how this can serve as a welcome counterpoint to what is being promoted today. As Glick (1992, p. 558) writes with respect to Werner’s theoretical orientation, “In some respects, Werner was a very modern thinker whose theoretical views were so at variance with normal professional practices that his message is yet to be heard.” I will argue, in a complementary manner, that Werner’s message about the conduct of research is one that should also be heard.

Werner received his doctorate in 1914, on the “Psychology of aesthetic experience”, and became assistant to Wilhelm Stern at the University of Hamburg in 1917—where he subsequently became Associate Professor (*Privatdozent* and then *Ausserordentlicher* Professor), up till 1933 (Valsiner 2005b, pp. 19–20). His book on the origins of metaphor (*Die Ursprünge der Metapher*) was published in Leipzig in 1919, by Felix Krueger of the Leipzig School of Gestalt^1^ (van Veer, 2005, p. 91). As van Veer (2005, p. 97) writes,

There is no doubt that Werner was well acquainted with the work of the Berlin Gestalt school—he repeatedly quoted the work of Koffka, Köhler, and others—but when it comes to influence, it is fair to say that he took more from the Leipzig *Ganzheitspsychologie* approach of Felix Krueger, Hans Volkelt, and Friedrich Sander. The two Gestalt schools shared many assumptions, e.g. an inclination towards phenomenology and a distrust of purely empiricist views, but the work of Krueger and his colleagues was explicitly developmental….

His subsequent book, *Einführung in die Entwicklungspsychologie*, first published in German in 1926, resurfaces in the more refined and developed exposition of his *Comparative Psychology of Mental Development* (Werner, 1948), published in English, with Werner’s transition to America. From 1933 till 1946, Werner held various temporary positions (at the level of Lecturer!) in American academic institutions. Finally, in 1947 he was appointed Professor at Clark University, and it was there that Werner established his organismic-developmental school of thought (Toomela, 2026).

Five years before his death, Werner presented a paper at a conference on the relationship between rehabilitation and psychology, held at Clark University. That paper was subsequently lightly edited and included in volume 2 of his selected writings (Werner 1978b). And it is there where he writes the following (p. 331):

Twenty-five years ago, when I became involved in the training of American students in experimental psychology, I got rather apprehensive at finding that students were frequently taught that there was only one acceptable kind of conduct in the laboratory: There had to be a hypothesis—or a set of hypotheses—rigidly set up, and the main job of the experimenter was to prove or disprove the hypothesis. What is missing here is the function of the scientist as a discoverer and explorer of unknown lands rather than only as a deductive methodologist.

Various authors (Crosby, 2001; Franklin, 1990; Valsiner, 2000; Witkin, 1965) have cited the paragraph from the unpublished copy, which was printed in 1962. For Franklin (1990, p. 185) the quotation reflects Werner’s particular “view of experimental inquiry”, while for Witkin (1965, p. 317) this expresses Werner’s “flexible approach to method”. For Crosby (2001, p. 45) the quotation suggests that Werner “was more interested in ‘probing’ than in ‘proving’”, while for Glicksohn (2025, p. 2) the quotation indicates Werner’s emphasis on “the *process* of investigation, and not the result of a hypothesis test.” Valsiner’s (2000, p. 82) reflection on this quotation is that “rigidity of thought has no place in science”. My four goals in this paper are: (1) to both explicate this quotation from Werner, and (2) to consider what could have led to Werner’s apprehension regarding the research culture that he encountered; (3) to consider why there has been resistance to Werner’s approach to research, and (4) to consider how Werner’s thinking here in the quotation still has relevance for active researchers of today, 65 years later.

Given that Werner’s talk was in 1959, his reference to a date twenty-five years earlier pinpoints this as being the year 1934. What do we know about that year in Werner’s life? In 1933, his position in the Psychological Institute at Hamburg University was abruptly terminated by the Nazis; subsequently, Walter Pillsbury, the Chair of the Department of Psychology at the University of Michigan (Raphelson, 1980) extended an invitation to him to join his faculty (Witkin, 1965, p. 310). Werner became a valued member of the staff (Raphelson, 1980, p. 309) during the three-year period of 1934–1936. So, what was going on there that would make him “rather apprehensive at finding that students were frequently taught that there was only one acceptable kind of conduct in the laboratory”?

During those years, Werner remained productive, publishing his first major paper in English in 1935 in the *American Journal of Psychology* (Werner, 1935) on contour formation. A contour produces a shape: “When the field is divided by a contour into figure and ground, the contour shapes the figure only, the ground appears shapeless” (Woodworth & Schlosberg, 1954, p. 411). Contour formation refers to the *process* of giving shape to a figure, and Werner presented the results of his study on that process in this paper, which has been cited to date 435 times, including most recently by Albertazzi (2024). The paper was discussed in the major texts on perception of the 1970 s (Haber & Hershenson, 1973, p. 198; Murch, 1973, p. 98); Werner’s (1935, pp. 42–43) explanation of the results of his series of 31 (!) experiments on contour formation was endorsed by Neisser (1967) in his book *Cognitive Psychology*, which defined the *second* cognitive revolution in psychology—the first being that of Gestalt psychology (Glicksohn & Lipperman-Kreda, 2007, p. 290).^2^ Neisser (1967, pp. 26–27) writes “It is fair to say that the phenomenon is still not well understood, but for the present we can keep Werner’s interpretation as a working hypothesis. Complete figure formation can take 100 msec. or more and is especially vulnerable in some of its later phases.”

Notably, Haber and Hershenson’s (1973) discussion of Werner’s results and interpretation appears in a section of their text entitled ‘microgenetic development of contours’. *Microgenesis* (i.e., microdevelopment) is Werner’s (1956) English term for the German *Aktualgenese*. Werner (1978a, pp. 111–112) presents a clear exposition of this:

In both microgenesis and ontogenesis the formation of percepts seems, in general, to go through an orderly sequence of stages. Perception is first global; whole qualities are dominant. The next stage might be called analytic; perception is selectively directed toward parts. The final stage might be called synthetic; parts become integrated with respect to the whole. Initially perception is predominantly ‘physiognomic.’ The physiognomic quality of the object is experienced prior to any details. At this level, feeling and perceiving are little differentiated. Again, in the early stages of development imaging and perceiving are not definitely separated.^3^

Proponents of the information-processing paradigm in cognitive psychology view the microgenetic school as something of a founding parent (Haber, 1969, 1974). However, as Glicksohn (2008, pp. 243–244) has stressed, “there is a clear distinction between the two: The information-processing conception is clearly mechanistic and atomistic (Leahey, 1992) and somewhat never-ending (cf. Neisser’s, 1976, p.17, processing — more-processing —even-more-processing caricature), while microgenesis is organismic and developmental in orientation (Werner, 1948).” In contrast, Haber (1969, pp. 3–4) argued that “Werner and his co-workers at Clark University have been most influential in arguing for and demonstrating this form of nonimmediacy of perception. Their model has not been as productive as it could be, primarily, I think, because it fails to provide very many operations to examine the changes occurring between stimulation and response. This is one reason why I think information-processing analyses are in a position to make for more rapid advances.” However, an information-processing analysis of creative thinking leaves much to be desired (Glicksohn, 2025), because such an analysis considers an *end-structure* of a process, and works backwards to retrospectively inform us of the supposedly sequential, linear progression of information processing, as depicted in the proposed model of the creative process.

Werner’s (1935) paper was accepted for publication in October, 1934. He acknowledges that the experiments reported were conducted in the Hamburg Psychological Laboratory, but “later subjected to check” at the “University of Michigan, Psychological Laboratory” (p. 40), with the support of Professor John Shepard and Professor Burton Thuma. Thus, in this first paper of Werner appearing in English, he had safely made the transition from Hamburg to Michigan. Werner’s (1937a) subsequent paper on depth perception was actually a 127-page monograph, and there he acknowledged that these new experiments were all performed at Michigan, againassisted technologies were used during the preparation thanking Prof. Thuma for support. Clearly, then, Werner during his three-year appointment at Michigan was both highly productive, but also very much involved in promoting laboratory research.

## There was only One Acceptable Kind of Conduct in the Laboratory

Raphelson (1980) points to John Shepard, who together with the Chair, Walter Pillsbury, was one “of the department’s two leaders”, as having a major influence on the research climate of the department. Raphelson writes (p. 305):

Shepard appeared as such a severe and cogent critic that his students seemed to hesitate doing anything on their own for fear of receiving in turn the unsparing criticism he directed toward the work of others. And then again, he worked so hard and so long in his laboratory and accumulated huge amounts of data which were never published. Some of his students had visions of themselves working just as hard and getting no further than he did.

Given this, both Werner’s creativity in research as well as his Gestalt orientation would certainly make him “rather apprehensive” when he “became involved in the training of American students in experimental psychology”. Regarding the first point, and in contrast with Werner, Shepard did not promote creativity. As Raphelson (1980, p. 305) writes “His strong points, curiously enough, often had the effect on his students of stifling creativity and productivity.” Regarding the second point, in spite of Pillsbury’s own interest in Gestalt psychology—as Raphelson (1968, p. 32) writes, Pillsbury “went to Berlin in the summer of 1928 in order to learn more of the Gestalt psychology”—and in spite of the impact that Wolfgang Köhler had made on the department when he spent a week there in 1929 lecturing on Gestalt psychology (Raphelson, 1968, pp. 38–39), the views about both Gestalt psychology and Werner were mixed. Thus, Pillsbury (1933) published a critique of Gestalt psychology, arguing that “No one has seen a type develop and probably no experiments can be devised that would enable us to start with the undeveloped sense impressions and see them grow by separate trials into the finished percept that we call the object” (p. 493)—apparently being unaware of the microgenetic technique (Bachmann, 2000) advocated by Werner at the time.

Werner, on his part, was teaching courses in the department on “Gestalt Psychology (‘a survey of experimental facts upon which Gestalt Psychology is based’)^4^, Characterology (‘Theories and experimental facts in the organization of character’), Developmental Psychology of the Higher Mental Processes (‘A survey of the special and general laws of development as seen in the comparative study of the child, primitive man, animals and phases of psychopathology’), Genetic Psychology and Special Problems in the Psychology of Music” (Raphelson, 1968, pp. 48–49). In the end, however, as Raphelson (1968, p. 49) suggests, “there was in Werner the German influence. The department was heavily inbred with Michigan graduates and was not very sympathetic to other national trends much less foreign ones. Pillsbury was never favorably inclined toward Gestalt Psychology and Shepard, though very close to it on many topics, was still critical of the system.”

Interestingly, there is a parallel here with respect to Frederic Bartlett at Cambridge University, in England. Bartlett (1956, 1962) had also been inspired by the Gestalt psychologists in the 1920s. As he recalls (Bartlett, 1962, pp. 387–388),

The first occasion on which I met Professor Köhler was at the International Congress of Psychology which was held in 1922^5^ at the University of Oxford….Following the Oxford meeting, Professor Köhler and Professor Koffka came and stayed for a few days as my guest in St. John’s College at Cambridge… I do not think that any of us came to the end of that morning prepared to accept everything in Gestalt theory, but I do know that we were all deeply attracted and stimulated, and that what we heard that Sunday morning became the basis of much later discussion, and especially of experimental work in the Cambridge psychological laboratory.

And yet, as Ramachandran and Vertinsky (2024, p. 136) reveal, in a letter dated July 1936, Bartlett “warned particularly against the ‘non-Englishness of German gestalt psychologists and Freudian-type models’.”

Thus, while much has been written about the fact that “Werner’s approach was not only outside the mainstream of American psychology in the 1940 s, 1950 s, and early 1960 s, but in many ways opposed to it” (Franklin, 1990, p. 185), it is also the case that Anglo-American psychology, both then and now, has a problem with the ‘non-Englishness’ or non-American nature of Gestalt psychology, whether of the Berlin school of Köhler (Ash, 1995), or of the Leipzig/Hamburg schools, with which Werner was affiliated (Bachman, 2000, p. 59; Barten & Franklin, 1978, p. 3; Franklin, 1997, p. 481; Hardesty, 1976; Ostler, 2016, p. 233; Rosar, 2014, p. 240; Steckner, 2004, p. 210; Wagoner, 2023, p. 19; Weimer, 1974, pp. 251–252). In fact, as Furnham (2017, p. 2) quips, “Americans have no idea there are any European psychologists.”

## The Main Job of the Experimenter was to Prove or Disprove the Hypothesis

It is instructive to return to Werner’s (1935) study. The basic experiment (experiment 1) comprises the presentation of a black disk for a very brief interval (e.g., 20 msec) on a white background, and after a short ‘pause’ (Werner’s term) [i.e., a brief interstimulus interval (ISI); e.g., of 220 msec] this is followed by a concentric black ring on a white background, appearing for a very brief interval (e.g., 20 msec)—and this sequence is repeated after a pause of between 280 and 560 msec. The inner diameter of the ring matches perfectly the diameter of the disk. In this situation, the ring disrupts the development of the disk’s contour. Phenomenologically, the perception is one of a dimmer disk having an indistinct contour. Werner notes in his lecture of December 17th, 1948, in his course on experimental psychology, that the optimal condition for the experiment has the above-noted exposure times and ISIs, and that “under these conditions, most observers find that [the disk] disappears and all that is seen is the flashing of the ring” (Werner & Wapner, 1948). Furthermore, “the greater strength of the ring figure doesn’t get to manifest itself until it has been presented two or three times”, and that this is because “time is needed to establish a relationship between [the disk] and [the ring]”.^6^

Table 1 presents a list of the first 11 experiments (of the 31 described in Werner, 1935) that were completed following experiment 1, the one which is cited in the textbooks (i.e., a black disk and a black ring). There is no exact replication of experiment 1, rather a whole series of constructive replications that, in Crosby’s (2001) terms ‘probe’ rather than ‘prove’.^7^

**Table 1:** Eleven consecutive experiments of Werner’s (1935) study

Experiment	Condition	Main finding
2	black and white reversed	same effect
3	the sequence is reversed (from disk-ring to ring-disk)	nullifies effect
4	circular figures are replaced by squares	same effect
5	irregular, angular figures are used	same effect
6	the disk is now made up of black and white spots	same effect
7	the black disk is made smaller	nullifies effect
8	the width of the ring is made smaller	same effect
9	the sizes of the disk and ring are altered	there is a rivalry between a black disk and a white disk
10	a half-ring is used	same effect
11	an incomplete figure is used	weaker development of contour
12	the disk appears with radiating spikes	same effect

Werner’s (1935) study *explores* the *phenomenon* of contour formation,^8^ and does not *test* a particular *hypothesis*.^9^ To my mind, this seems to be a better use of the experimenter’s time, effort, and resources than in setting up a series of specific hypotheses (today, in a pre-registered study), which by definition limits the scope of the experimenter’s study.^10^

Let me consider a paper published by Glicksohn (1992) to further consider this distinction between the systematic exploration of a phenomenon, so admirably exemplified by Werner’s (1935) study, and the explicit goal set out in that paper, of trying to “prove or disprove the hypothesis” that “the more varied the sensory environment (i.e., perceptual overload versus perceptual deprivation), the longer the time that is verbally estimated, and the shorter the time estimation obtained by the method of production” (Glicksohn, 1992, p. 634). I shall focus here on the creation of these altered sensory environments in that study, and specifically on the definition of *perceptual deprivation* and *perceptual overload*, as employed in that study.

Perceptual deprivation can be defined as exposure to a diffuse, unpatterned, monotonous, homogeneous sensory environment, namely a *Ganzfeld* (Avant, 1965). Operationally, in this study such a *Ganzfeld* was created by first placing two halves of a table-tennis ball over the eyes, secured via hypoallergenic surgical tape, and then exposing the participant to red light emanating from seven 25-watt light bulbs, that were placed behind individual milky white plastic covers that helped to make the red light diffuse. The lights were positioned above, in front of, and to the sides of the participant. The primary source for this procedure was the study of Hochberg et al. (1951).^11^ While this condition of perceptual deprivation is primarily determined by visual underload, one can also concomittantly expose the participant to auditory underload, such as white noise (Glicksohn, 1992), brown noise (Kübel et al., 2021), or pink noise (Miskovic et al., 2019)—this use of underload in both modalities is referred to as a *multimodal Ganzfeld* (MMGF) in current studies (Miskovic et al., 2019; Pütz et al., 2006).^12^

In contrast to perceptual deprivation, Glicksohn (1992, p. 637) coined the term ‘perceptual overload’ referring to “different stimulation [which] is monotonously presented (e.g., flashing lights, having a constant interstimulus interval, in the visual field)”. Operationally, such a condition was created in the lab by exposing the participant to a battery of four flashing 25-watt coloured light bulbs, and two slide projectors projecting two coloured slides in parallel that changed automatically every 15 s. The primary source for this procedure was the study of Zuckerman et al. (1970), secondary sources being Ludwig (1971, 1972); others had previously used an immersive sensory overload *space* (e.g., Haer, 1971).^13^

To create a condition of auditory overload, a dichotic presentation to the two ears of different selections of similar monotonous music was employed (Glicksohn, 1992).^14^ The experimental design was a 3 (levels of visual stimulation: *Ganzfeld*, visual overload, control) × 4 (levels of auditory stimulation: white noise, auditory overload, stereophonic presentation of monotonous music, no auditory stimulation) factorial one.^15^ As a working hypothesis,^16^ the *Ganzfeld* coupled with white noise was viewed as constituting perceptual deprivation, and visual overload coupled with auditory overload as constituting perceptual overload.

I believe that Werner would view this study as aligning with his own organismic, sensory-tonic theory of perception (Werner & Wapner, 1952), in that exposure to an altered sensory environment is expected to have a marked influence on the organism, thus impacting on time perception—much as he found in his own studies (Denner et al., 1963; Langer et al., 1961). His colleague in Michigan, John Shepard, however, would probably not have been convinced. As Raphelson (1980, p. 305) writes, “Shepard would pick out the many neglected controls in the experiments that dominated the literature and convince his listeners that he was one experimenter who knows what precautions to take.” Here is a plausible list of four concerns that he might have raised:

1. What is the optimal time of exposure to an altered sensory environment to ensure that the conditions do consitute those of perceptual deprivation and perceptual overload?^17^
2. For the condition of visual overload, what is the optimal flash rate of the coloured light bulbs? Why was a regular presentation of flashes used? Furthermore, there was no systematic investigation of the effects of different flash rates in this study.^18^
3. For how long after exposure to an altered sensory environment can one assume that the prior stimulation will still have an aftereffect, or persist?^19^
4. Why was the time-production task completed *after* rather than *during* exposure to an altered sensory environment?^20^

Given this list of things to consider, the researcher might still be piloting the study (as Shepard’s students probably found themselves doing). That is, if the goal in that study was to unconditionally “prove or disprove the hypothesis”. In contrast, as Werner (1978b, pp. 331–332) suggested, “To be sure, hypotheses are essential intellectual elements of inquiry, not, however, as rigid propositions, but as flexible parts of the process of searching.^21^ By the same token, conclusions drawn from results are as much a beginning as an end.” Let us now turn to consider Werner’s view of the “scientist as a discoverer and explorer of unknown lands.”

## Scientist as a Discoverer and Explorer of Unknown Lands

Werner (1978b, p. 332) writes:

… the experimental findings are exactly what Duncker (1945), in his analysis of problem-solving behavior, has termed “functional solutions”^22^ … Nowadays, I hopefully submit, “academic psychologists” begin to see research not as a rigid exercise of rules of a game, but essentially as a problem-solving procedure, a probing into unknown territories with plans which are not fixed but modifiable, with progress and retreat….

Thus, Werner is stressing that research implements a process of problem solving, one in which even when problems are resolved they inevitably lead to new problems which “suddenly emerge”.^23^ This focus on *process*^24^ rather than end-product, that Werner stressed in his writing when he spent the academic year at Harvard in 1936–1937 (Werner 1937c), is one which he evidently found lacking in the departmental climate at Michigan in 1934.

It is worth recalling that Werner’s academic position in America between 1934 and 1947 was precarious (Witkin, 1965, p. 311). His position at Michigan between 1934 and 1936 was as an adjunct professor, on ‘soft money’ (Raphelson, 1980). The year at Harvard as a visiting professor was as Gordon Allport’s replacement during the latter’s sabbatical. He finally secured a position matching his international standing, at Clark University as Professor in 1947 (Witkin, 1965). Throughout this period of academic and financial instability, attempts were made to find him an academic home. Winston (2010, p. 102) cites a latter from Harvard’s Chair, Edwin Boring, to Kurt Lewin, written in 1937: “I am distressed and worried about Werner …. what in the world am I to do about Werner, since there is no other job for him here when Allport shall have come back and resumed his entire salary?” Allport, in turn, was enthusiastically trying to find a position for his friend, Heinz Werner, but even as late as 1944 he was still writing a letter of recommendation for Werner for the position of *Instructor* (!) at Brooklyn College. He writes (Allport, 1944):

In 1923, I attended his lectures at the University of Hamburg, and since that time have been frequently in contact with him. During 1937, he took my place at Harvard while I was on leave.

His scientific work seems to me to stand in the highest rank. He is a productive experimentalist, and has a wide range of fields: psychophysics, language, thought processes, mental development…. His sphere of competence is unusually broad.

Werner’s resilience during these years is remarkable.

Werner’s emphasis on *process*^25^, rather than product or achievement (Werner 1937c) remains relevant 65 years later. Notably, in his foreword to a book addressing impulsivity, Loewenstein (2010, pp. xv-xvi) returned to this notion of scientific research being “like a voyage of discovery into unknown lands, seeking not for new territory but for new knowledge” (p. xi), and continues: “after making real scientific progress and feeling on the verge of closing in on the answer to one’s central question, the very process of discovery provides an often abrupt increase in one’s appreciation of the complexities of the topic. The knowledge gap between what one knows and what one wants to know widens abruptly.” This suggests that a “discoverer and explorer of unknown lands” has to move out of their comfort zone. This, in turn, resonates with the sentiment expressed by Mayer (1995, p. 26) that “the Gestalt psychologists work on *hard* questions, whereas modern cognitive psychologists sometimes prefer *easy* ones.”

To return to the title of this paper: Indeed, *quo vadis*? In this day and age characterized by a concern regarding the ‘replication crisis’ (with its accompanying problems; Bird, 2021; Maxwell et al., 2015)^26^ and the complementary advocacy of the pre-registration of studies (with its own potential problems; Logg & Dorison, 2021; Yamada, 2018)—where both replication and pre-registration have the goal of convincing the reader that the experimenter can reliably “prove or disprove the hypothesis”—the emphasis will be on the end-product and not necessarily on the process. Indeed, proponents of pre-registration would presumably argue that an exploratory approach, such as employed in Werner’s (1935) study on contour formation, is important, but this must be followed by a pre-registered confirmatory study. A strong argument has been made by Simmons et al. (2021) who believe that as pre-registration “becomes increasingly popular”, so “reviewers and readers should be naturally circumspect about any experiment that is reported without a pre-registration” (p. 159), because that type of experiment (such as Werner’s) “can possibly coast on the ambiguities of reporting standards from an earlier era” (p. 159). Of course, even if “experiments are well-suited to preregistration because every aspect is controlled by the experimenter” (Simmons et al., 2021, p. 160), it is still the case that outliers might be found in the data. Given the existence of outliers, one approach here, advocated by Wichmann and Hill (2001, p. 1310), is “to gather additional data at stimulus intensity … before more radical steps, such as removal of [the outlier] … from one’s data set, are considered.” The problem here is that pre-registration does not mesh well with the deletion of outliers from one’s dataset.

Indeed, as noted above, if the experiments are viewed as “situations to generate hypotheses and ‘explore’ the complexities of some phenomena through the participant’s constructive responses to the experimenter’s manipulations” (Wagoner, 2009, p. 112)—such as in the case of Werner’s (1935) study—then pre-registration will simply be detrimental to such a goal. Furthermore, if “research is understood primarily as the production of effects, phenomena, and events” (Derksen & Morawski, 2022, p. 1491)—again, as in Werner’s study—then it is important to “work through the protocols in the hope of finding something interesting or being able to systematize problem solutions” (Jäkel & Schreiber, 2013, p. 26).

Opponents of pre-registration would presumably argue that Werner’s (1935) exploratory approach is the one to be adopted, because, as McDermott (2022, p. 56) writes, “*preregistration* will hinder our ability to wonder about the world and discover the previously unknown in ways that are scientifically limiting as well as downright sad.” Indeed, as Mazur (2020, p. 616) argues, “in practice hypothesis testing often overshadows genuine questions in psychological research.” Valsiner (2000, p. 61) cautioned us twenty-five years ago about this:

Such modern reliance on the ‘standardized methods’ is akin to medieval scholasticism (with inquisition-like testing of the ‘solidity of scientific character’ through examination of whether ‘the right’ methods are used by the scientists). Nothing can be more productive for stopping active and open enquiry into the nature of phenomena of any science.

Consider, then, a researcher attempting a constructive replication of Werner’s (1935) study today, who for example would hypothesize for experiment 6 (see Table 1) that it is the size of the black and white spots that will affect the results. Or, perhaps, their number/density. Or, perhaps the ratio of black to white. All of these variations on a common theme would then be pre-registered. In line with that, for example, consider the type of detail required to pre-register experiment 12. Exactly how many radiating spikes? Is the hypothesis here that with an increase in the number of radiating spikes, the effect becomes stronger (or, weaker)? Must they be evenly spaced? Consider, further, the fact that without having run all or most of these 31 experiments, in order to see what happens, it would be hard to justify the pre-registration of this study. The Wernerian notion of a developmental and inherently interactive process, with its “one step backward in order to take two steps forward” (Kaplan et al., 2005, p. 135) can well guide us in both understanding Werner’s *Gestalt*-oriented thinking here, and in adopting such a process-oriented, open-ended approach in our own research.

## Data Availability

No datasets were generated or analysed during the current study.

## Footnotes

1 As Weimer (1974, pp. 251–252) writes, “Although it is not well known in America, there were really two major variants of Gestalt psychology. The more familiar group, the Berlin school led by Wertheimer, Koffka and Köhler, emigrated almost *in toto* to America … The other Gestalt theorists, the Leipzig school led by Felix Krueger (Wundt’s assistant and then successor at Leipzig), Karl Sander, and Hermann Volkert, were genetic psychologists in the organismic-developmental tradition. These theorists referred to their position as *Ganzheit* (holistic) psychology. The developmentalist Heinz Werner epitomized this tradition in America and was one of the few writers to cite the Leipzig school.” Indeed, Werner (1937b) specifically writes that “Felix Krueger is doubtless one of the leaders in modern Gestalt psychology.” However, as Wagoner (2023, p. 9) stresses, “Werner was never a member of the Leipzig faculty but was a participant in an early experiment there that used the method of *Aktuelgenese* … which Werner … later transformed into ‘microgenesis’.” This *Ganzheitspsychologie* influence on Werner was, as Valsiner (2005a, p. 395) suggests, “fortified by the Hamburg tradition (William Stern, Ernst Cassirer, and Jakob von Uexküll).”

2 Uljana Feest questions what is *cognitive* about Gestalt psychology, and whether Werner was a player in the first cognitive revolution. If by *cognitive* one is referring to a computer analogy of ‘information processing’, then what is the Gestalt take on such an analogy? As Henle (1987, p. 18) forcefully wrote, “Why not, for example, integrate psychology by means of [the] computer model, which has so many advocates today, and whose terminology has crept into the vocabulary of almost every psychologist and of nonpsychologists as well? What is relevant … is that a computer model is still a mechanistic model, though a highly sophisticated one; it achieves order by means of existing hardware and software, not by free dynamics.” Henle (1977, p. 4; 1989, p. 81) is not the only Gestalt theorist who views artificial intelligence (AI) as yet another manifestation of a ‘machine theory’. Indeed, for Arnheim (1999, p. 182), “The present practice of Computers is far distant from the gestalten that solve high tasks of problem solving”, while for Wertheimer (1985, p. 22), “A blind or mechanical application of schema-generalization is not apt to be productive; the restructuring and insight emphasized in Max Wertheimer’s book are still missing in the contemporary models.” This is the same point made by Dreyfus (1967, p. 22) early on (see also Dreyfus, 2012), who writes: “What is lacking is any way of organizing the problem so that one can see which operations are significant and which trivial. Wertheimer, in his classic work *Productive Thinking*, points out that the associationist account of problem solving excludes the most important aspect of problem-solving behavior, viz., a grasp of the essential structure of the problem, which he calls ‘insight.’ In this operation one breaks away from the surface structure and sees the basic problem—what Wertheimer calls the ‘deeper structure’—which enables one to organize the steps necessary for a solution.” Not unexpectedly, a major proponent of both Information Processing and AI expressed his own views on the Gestalt approach to creativity (Simon, 1986, pp. 253–254): “I do not know for certain whether Gestalt theory is compatible with contemporary information processing theory of human mental functions. I have always thought that it probably is, provided an honest attempt is made to interpret sympathetically the terms of Gestalt theory that are not provided with adequate operational definitions…. I will regret it a bit if the colorful vocabulary of Gestalt psychology is ruled out of bounds for information processing psychology, for fine words like ‘intuition,’ ‘insight,’ ‘understanding,’ and even ‘Gestalt,’ can surely be sharpened up and made useful to experimental psychology. But if Gestalt psychologists persist in their claim that we are twisting these terms out of all semblance of their intended meaning, it may be simpler to forego them and replace them by others…. There should be no mistake, however, about the consequences of such a decision. The final test of a psychological theory is whether it fits behavior. If information processing psychology explains the phenomena, as it increasingly does, then we are obliged to reject any interpretation of Gestalt theory that insists on distancing itself from these empirical tests.” Indeed, Simon (1999, p. 3) readily acknowledges the importance of Duncker’s work to his own information-processing approach to problem solving, but further marks Selz as the main theorist here (see also Newell et al., 1958, p. 164), and his notion of ‘schematic anticipation’. If for Simon (1986), the Gestalt approach to thinking and creativity is ‘colourful’ (i.e., fuzzy), and does not provide a better fit to the data than does that of Information Processing, for Arnheim (1999) and Wertheimer (1985) this good fit of Information Processing is to the modelling of well-defined problems, that do not require restructuring and insight—that is, to (reproductive) problem solving, but not to ill-structured problems that do require productive thinking (Öllinger & Goel, 2010, p. 19). In this respect, what is *cognitive* about Gestalt psychology is exactly what differentiates it from the dominant information-processing paradigm. Furthermore, Werner was certainly a player in this first cognitive revolution, as clearly seen in his major publications appearing before Neisser’s (1967) text, while being referenced by Neisser in that text—including a reference to Werner (1948).

3 As Flavell and Draguns (1957, p. 197) write, microgenesis refers “to the sequence of events which are assumed to occur in the temporal period between the presentation of a stimulus and the formation of a single, relatively stabilized cognitive response (percept or thought) to this stimulus. More specifically, the term will refer primarily to the prestages of extremely *brief* cognitive acts, e.g., the processes involved in immediately perceiving a simple visual or auditory stimulus, conceptually generating a word association, etc. … The paradigm of microgenesis of perception … usually entails the successive presentation of a stimulus under conditions of increasing clarity. Successive tachistoscopic presentation of visual stimuli with exposure times gradually increasing until complete perception is possible, considered as the experimental homologue of the everyday, near-instantaneous process of simply ‘seeing’ an object, would perhaps be the best example of this paradigm.”

4 As Barten and Franklin (1978, p. 3) write, Werner’s “organismic-developmental approach distinguished him from the Berlin Gestaltists with whom he shared an opposition to elementaristic psychology. He always felt himself more closely allied with the organismic holism of the Leipzig school of Krueger, Volkelt, and Sander, and was to develop Sander’s … concept of *Aktualgenese* in his work on microgenesis.”

5 Bartlett’s memory fails him here. As noted by Goodblatt and Glicksohn (2022, p. 9), “A major event occurred in 1923. This was the *Seventh International Congress of Psychology*, which was held at Oxford University (Langfeld, 1924). As a result of World War 1, that there had been very little contact for about a decade between German psychologists and their colleagues in the English-speaking world. At this conference, Koffka spoke in English, describing his work (Langfeld, 1924: 151).”

6 A former student of Werner (Cirillo, 2005, pp. 229–230) writes: “Werner’s course in experimental psychology, which all first-year graduate students attended, was a treat for me. Configurationist and developmental principles were embodied in empirical studies.” Werner’s (1935) study on contour formation exemplifies this integration of configurationist and developmental principles.

7 I thank Uljana Feest for bringing to my attention the literature in the philosophy of science addressing ‘new experimentalism’ (Feest & Steinle, 2014; Hacking, 1983) or ‘exploratory experimentation’ (Feest & Devezer, 2026; Steinle, 1997), that help to situate Werner’s emphasis on exploration and discovery in this “broader philosophy of science context”. Werner’s (1935) systematic study of the process of contour formation (see Table 1), entailing a series of constructive replications, brings to mind the series of experiments conducted by Ampère a century earlier, studying the effect of an electric wire supported by two variable posts on a magnetic needle supported such that it could rotate only in a plane perpendicular to the Earth’s magnetic field. As Steinle (1997, pp. S66-S67) writes, “As a result of the experiment, Ampère formulated the general rule that the needle always swings into a position perpendicular to the direction of the wire. This experiment was the endpoint of a whole series of experiments in the course of which the relative position between the needle and a nearby ‘connecting wire’ was systematically varied. The goal was to discover a rule giving the behavior of the needle.” The ‘new experimentalism’ emphasizes what was central to Werner: “processes of discovery and theory construction” (Feest & Steinle, 2014, p. 276), and the “notion of *exploratory research* that highlights two crucial features: the systematic variation of experimental and model parameters on the one hand and the conceptual openness of the research situation on the other” (Feest & Devezer, 2026, p. 12). Indeed, as Tong (2019) has suggested, “most scientific research is exploratory in nature: the design, conduct, and analysis of a study are necessarily flexible, and must be open to the discovery of unexpected patterns that prompt new questions and hypotheses. In this context, statistical modeling can be exceedingly useful for elucidating patterns in the data….”

8 Werner was clearly a rigorous experimenter, and at the same time was also less rigid than his American colleagues. Hence, being less rigid does not imply being less rigorous (my thanks to Uljana Feest for this point). Furthermore, his exploratory experimentation is far from being one of random exploration—it is one exemplifying an “iterative process of trying to establish empirical regularities and mechanisms that are connected to a given object of research, to delineate the boundaries of that object, and to provide theoretical descriptions of them” (Feest & Devezer, 2026, p. 8), and of “aiming to provide a carefully planned, systematic mapping of unknown spaces” (Feest & Devezer, 2026, p. 5).

9 Indeed, Werner’s study exemplifies a ‘good’ experiment, “from a Gestalt psychological perspective” (Feest, 2019, p. 11), namely: “one that reliably evokes the relevant phenomenal experience, thereby giving rise to robust experimental results.”

10 Note that Werner stresses the epistemic process of research as one of *exploration* as opposed to hypothesis-testing, though of course hypothesis-testing plays an integral part of this epistemic process. Werner’s (1935) study on contour formation reveals the process of forming that percept, as does his other work on the microgenesis of perception (Werner 1978a). But the epistemic process of research *is* related to the microgenesis of perception. Thus, for example, in our own work using a microgenetic technique (Yaniv & Glicksohn, 2021), we are more interested in the participants’ reports of their unfolding perceptual experience, than in requiring those participants to make speedy judgments (e.g., for the detection of a particular feature in the image presented).

11 Rock et al. (2008) have replicated this procedure; others have used white light (Lloyd et al., 2012) and green light (Kübel et al., 2021).

12 In more recent studies, participants have been exposed to a *Ganzfeld space* (i.e., without table-tennis balls or goggles) that provides a more immersive experience (Glicksohn et al., 2017, 2019; Pistolas & Wagemans, 2025; Pistolas et al., 2025).

13 The definition of perceptual overload is quite problematic. Wohlwill (1974, pp. 131–132) writes: “There appears to be at least three major aspects worth differentiating: intensity, diversity, and patterning.” Lipowski (1975), in turn, notes that “the term *sensory overload* connotes for some writers a state occurring in response to ‘intense multisensory experiences,’ or one in which two or more sensory modalities are stimulated at higher than normal intensity and this combination of stimuli is introduced suddenly.” More recently, Scheydt et al. (2017, p. 114) suggest that one should distinguish between “sensory overload as a situation in which excessive or atypical stimulation occurs … and as a state resulting from the reaction to a stimulating situation.” Note that the same distinction between situation (or condition) and state applies to perceptual deprivation.

14 I am not familiar with any follow-up by others using this technique. Note that as opposed to the dichotic presentation of music to one ear and white noise to the other (Hae & Hatin, 2023), here we have similar but different music presented simultaneously to both ears.

15 Auditory stimulation was presented via headphones. Others have employed loudspeakers or tape-recorders, placed around the participant in the lab (Wohlwill et al., 1976; Zuckerman et al., 1970).

16 Uljana Feest suggests that by using the term *working hypothesis* I mean to indicate “the temporary character of such hypotheses within a conception of research that focuses on the process rather than the end product.” I certainly agree, and further note that such an understanding is aligned with Werner’s (1937c) own emphasis on studying the process and not just the end product (see Glicksohn, 2025, for a relevant discussion). Let me add here a reference to Kaplan (1964), who writes (p. 88): “To carry forward an inquiry we may formulate working hypotheses. These serve to guide and organize the investigation, providing us something to go on with. The working hypothesis is not a guess at the riddle, a hunch as to what the answer might be. It is an idea, not about the outcome of inquiry but about the next steps that may be worth taking. The working hypothesis formulates a belief pertaining to the course of inquiry but not necessarily pertaining to its ultimate destination.”

17 In this study, exposure time was 20 min, prior to task performance. Others have used 10 min (Miskovic et al., 2019), 15 min (Rock et al., 2008), 20 min (Pütz et al., 2006), 25 min (Pistolas & Wagemans, 2025), 30 min (Lloyd et al., 2012; Wohlwill et al., 1976), 45 min (Haer, 1971), 8 h (Zuckerman et al., 1970) and beyond that (Suedfeld, 1980).

18 Glicksohn and Weisinger (2022) have recently reported on a parametric analysis of flicker rate on time production, in a study employing visual flicker, auditory flicker, and audio-visual flicker. A main finding in this study was that time production was veridical at a flicker rate of 6 Hz, longer time productions being found for flicker rates up to this, and shorter time productions being found for flicker rates higher than this (at 10 Hz an asymptotic value is achieved). In this study, time production was completed *while* being exposed to flicker.

19 Frankenhaeuser and Lundberg (1974) reported a delayed effect of noise on task performance, further suggesting that noise could have a positive effect on the performance of boring tasks.

20 In the Glicksohn and Weisinger (2022) study, time production was completed *during* exposure to an altered sensory environment of flicker, while in the Glicksohn et al. (2017) study, time production was completed *after* exposure to an immersive, whole-body perceptual deprivation environment. The problem with completing the task of time production while being exposed to an altered sensory environment, such as that of perceptual deprivation, is that such a task (in fact, any task) might well disrupt the effects of such an environment (Suedfeld, 1980, pp. 67–68). Thus, time production *following* exposure to perceptual deprivation “is not necessarily a limitation of the present study. This, however, is an issue worth considering in future studies in this domain” (Glicksohn et al., 2017, p. 8). Incidentally, the same is the case with respect to task performance during an aesthetic experience, that will also derail that experience (Cupchik et al., 2009, p. 87).

21 By “flexible parts of the process of searching”, Werner might well be referring to the notion of “schematic anticipation” proposed by Otto Selz (1881–1943). Schematic anticipation relies on the existence of a schema (itself being a *gestalt*) which guides problem solving. As ter Hark (2004, pp. 99–100) elaborates, Selz realized “that in many cases of memory retrieval subjects already know that the information at hand is a piece of a larger structure. Indeed, they even often know to what kind of structure the piece belongs. Being aware of this relational structure prompts the subject to anticipate schematically the answer to the question.” Thus, being aware of the whole structure of the problem enables one to realize where the gap in knowedge rises, problem solving hence being a process of gap-filling. As Humphrey (1951, p. 140) summarizes, for Selz “the problem is a set of data with a gap; and the effect of the determination is to initiate previously applied methods to fill the gap.” Selz’s work was both slighted and then unacknowledged. Slighted, because his work on thinking (Proctor & Ridderinkhof, 2022) was viewed by the Berlin school of Gestalt psychology as reflecting a ‘machine theory’ (Proctor & Ridderinkhof, 2022, p. 2358; ter Hark, 2010, p. 19) —one that was mechanistic, “that sought to reduce goal-directed and meaningful events to a sum of, in itself, meaningless parts of events” (ter Hark, 2004, p. 210). Unacknowledged, because although this work was, in fact, cited by the Gestalt psychologists (e.g., Duncker, 1945, p. 8; Koffka, 1935, p. 560), it was not accepted as part of the Gestalt approach to productive thinking. Selz, in contrast, did consider his theory as being in line with Gestalt theory (Proctor & Ridderinkhof, 2022, p. 2358; ter Hark, 2004, p. 94, p. 210), though one which suggested that “Gestalt psychology overlooked the implications of a theory of schematic anticipations for a bottom-up approach” (ter Hark, 2010, p. 20). If for Werner, a hypothesis can serve as a flexible part of the process of searching, then in line with Selz, this would suggest that the hypothesis attempts to fill the gap in one’s inquiry by providing a specific prediction *qua* schematic anticipation. But, as Werner further suggests in this quotation, as one gap is solved, another opens up (“conclusions drawn from results are as much a beginning as an end”).

22 Duncker (1945, p. 29) had written that the “decisive points in thought-processes, the moments of sudden comprehension, of the ‘Aha!,’ of the new, are always at the same time moments in which such a sudden restructuring of the thought-material takes place, in which something ‘tips over’.” Insight, then, is when “Parts and elements of the situation which, psychologically speaking, were either hardly in existence or remained in the background—unthematic—suddenly emerge, become the main point, the theme, the ‘figure’” (Duncker, 1945, p. 29). Thus, restructuring of the problem situation results in insight.

23 As Wagoner (2009, p. 112) writes, “experiments can be conceived as situations to generate hypotheses and ‘explore’ the complexities of some phenomena through the participant’s constructive responses to the experimenter’s manipulations. This is particularly true in the study of processes and development.”

24 For Werner, this process is a *developmental* one that conforms to his *orthogenetic principle* “which states that wherever development occurs it proceeds from a state of relative globality and lack of differentiation to a state of increasing differentiation, articulation, and hierarchic integration” (Werner 1978a, pp. 108–109). However, as Toomela (2026, pp. 116–117) has argued, this orthogenetic principle was viewed by Werner as being axiomatic, hence not testable. As Toomela writes (p. 115), “Developmental processes of differentiation and hierarchical integration turn out to be just statements with no explanatory power, they just ‘happen’.” In order to make the orthogenetic principle testable, he suggests the following (p. 133): “A whole changes when its parts and/or the relationships between the parts change. Change of a whole, without change in parts or in the relationships between the parts, must be impossible. These are empirically testable assumptions.” I wonder whether in his lecture notes discussing the study on contour formation referred to earlier, Werner was cognizant of this, for, as noted earlier, he states there that “time is needed to establish a relationship between [the disk] and [the ring]”. Valsiner (2026, p. vi) suggests that the parts of the whole should have “inherent direction in which … [they are] open to transformation.” Note that such inherent “intentional directionality” (Valsiner, 2026, p. vi) imparts a *teleological* theme to the developmental process.

25 As Glick (1992, p. 561) writes, “From the Wernerian point of view, the process will not simply reveal itself either by close examination or by a sensitive approach to data analysis. The process can only be revealed by a planned structure of investigatory activity that constantly seeks to find differentiating measurement operations that can expose to view process differences that might otherwise be hidden.” This emphasis on process is stressed by others who, like Glick, are also influenced by Werner, including Wagoner (2023, p. 16) and Raeff (2020, pp. 251–252). Glicksohn (2025) has recently discussed the implications of Werner’s (1937c) thinking for the study of creative thinking.

26 What would be Werner’s take-home message regarding replicability? Werner’s (1935) study on contour formation exemplifies the notion that it is not sufficient to conduct a process of exploratory experimentation; the study must also entail constructive replication (see Table 1) to be convincing. Indeed, it is better to provide evidence for internal replication within a study, than to leave this to others. For as Bem (2011, p. 421) writes “As all research psychologists know, many procedures are tried and discarded during this process. This raises the question of how much of this pilot exploration should be reported to avoid the file-drawer problem, the selective suppression of negative or null results…. More general issues of replication are also pertinent here. Although most academic psychologists do not believe in psi, many apparently do believe in miracles when it comes to replication.”
